# The role of mTOR and phospho-p70S6K in pathogenesis and progression of gastric carcinomas: an immunohistochemical study on tissue microarray

**DOI:** 10.1186/1756-9966-28-152

**Published:** 2009-12-13

**Authors:** Li Xiao, Yi C Wang, Wu S Li, Yan Du

**Affiliations:** 1 Department of Emergency, Shengjing Hospital of China Medical University, Shenyang, Liaoning, China; 2 Endoscopic center, The First Affiliated Hospital of China Medical University, Shenyang, Liaoning, China; 3 Department of Oncology, Shengjing Hospital of China Medical University, Shenyang, Liaoning, China

## Abstract

**Background:**

mTOR signaling pathway and its downstream serine/threonine kinase p70S6k were frequently activated in human cancers. The dysregulation of the mTOR pathway has been found to be a contributing factor of a variety of different cancer. To investigate the role of mTOR signal pathway in the stepwise development of gastric carcinomas, we analyzed the correlations between the mTOR and P70S6K expression and clinic pathological factors and studied its prognostic role in gastric carcinomas.

**Methods:**

mTOR and phospho-p70S6K proteins were examined by immunohistochemistry on tissue microarray containing gastric carcinomas (n = 412), adenomas (n = 47) and non-neoplastic mucosa (NNM, n = 197) with a comparison of their expression with clinicopathological parameters of carcinomas.

**Results:**

There was no difference of mTOR expression between these three tissues (p > 0.05). Cytoplasmic phospho(p)-P706SK was highly expressed in adenoma, compared with ANNMs (p < 0.05), whereas its nuclear expression was lower in gastric carcinomas than gastric adenoma and ANNMs (p < 0.05). These three markers were preferably expressed in the older patients with gastric cancer and intestinal-type carcinoma (p < 0.05). mTOR expression was positively correlated with the cytoplasmic and nuclear expression of p-P70S6K(p < 0.05). Nuclear P70S6K was inversely linked to tumor size, depth of invasion, lymph node metastasis and UICC staging (p < 0.05). Univariate analysis indicated that expression of mTOR and nuclear p-P70S6K was closely linked to favorable prognosis of the carcinoma patients (p < 0.05). Multivariate analysis showed that age, depth of invasion, lymphatic invasion, lymph node metastasis, Lauren's classification and mTOR expression were independent prognostic factors for overall gastric carcinomas (p < 0.05).

**Conclusion:**

Aberrant expression of p-P70S6K possibly contributes to pathogenesis, growth, invasion and metastasis of gastric carcinomas. It was considered as a promising marker to indicate the aggressive behaviors and prognosis of gastric carcinomas.

## Introduction

Gastric carcinoma ranks as the world's second leading cause of cancer mortality behind lung cancer despite a sharp worldwide decline in both its incidence and mortality since the second half of the 20th century. It continues to be a major health problem because of the slow decrease in incidence in Asia and high mortality of diagnosed gastric carcinoma in West [1]. Therefore, it is of much significance for the prevention, treatment and prognosis evaluation of gastric cancer to clarify its molecular mechanisms and find out a good biomarker to indicate its carcinogenesis and subsequent progression.

The mammalian target of rapamycin (mTOR) also known as FK506 binding protein 12-rapamycin associated protein 1 is a serine/threonine protein kinase that supports cell growth, cellular metabolism, cell proliferation, cell motility, cell survival, protein synthesis, and transcription such as angiogenesis and autophagy. mTOR that is an evolutionarily conserved serine-threonine kinase of a 289-kDa in length belongs to the phosphoinositide 3-kinase (PI3K)-related kinase family. mTOR is composed of an N-term; 20 tandem repeats-HEAT which are implicated in protein-protein interactions; and a C-term which includes a FAT domain, a FBR domain, a kinase domain, a NDR domain and a FATC domain. The FATC domain is essential to mTOR activity and the deletion of a single amino acid from this domain abrogates the activity. mTOR can be autophosphorylated via its intrinsic serine/threonine kinase activity. mTOR exerts its multiple functions in the context of two different multiprotein complexes: mTOR complex 1 (mTORC1) and mTOR complex 2 (mTORC2). mTORC1 is composed of mTOR, Raptor, mLST8, and PRAS40, and importantly activates p70 ribosomal protein S6 kinase and inactivates eIF4E binding protein 1, which promotes protein translation and cell growth. Conversely, mTORC2 is composed of mTOR, Rictor, Sin1, and mLST8, phosphorylates and activates another member of the AGC kinase family, Akt. Current research indicates that mTOR integrates the input from multiple upstream pathways, including insulin, growth factors (such as IGF-1 and IGF-2), and mitogens. mTOR also functions as a sensor of cellular nutrient and energy levels and redox status [2-5].

P70 S6 kinase (p70S6K) is activated in a signaling pathway that includes mTOR. P70S6K is a mitogen-activated Ser/Thr protein kinase that is required for cell growth and G1 cell cycle progression. This kinase is controlled by multiple phosphorylation events located within the catalytic, linker and pseudosubstrate domains and subsequently phosphorylates specifically ribosomal protein S6. Activation occurs via phosphorylation at ser411, Thr421 and Ser424 within the pseudosubstrate region. Phosphorylation of Thr229 in the catalytic domain and Thr389 in the linker domain are most critical for kinase function. Stimulation of mammalian cells by a variety of mitogenic stimuli results in a rapid, biphasic activation of p70S6K. Inhibition of p70 activity inhibits the entry into S phase of the cell cycle and exhibits cell cycle arrest at G0/G1 phase, suggesting that the activation of p70S6k plays an obligatory role in mediating mitogenic signals during cell activation [6-8].

mTOR signaling pathway and its downstream serine/threonine kinase p70S6k were frequently activated in human cancers and the dysregulation of the mTOR pathway is implicated as a contributing factor to various human disease processes, especially various types of cancer[5,6,8-11]. However, the mTOR/p70S6K signaling pathway in gastric carcinomas has not been investigated so far, which impel the authors to investigate the role of mTOR/p70S6K signaling pathway for finding a novel target for the anticancer drugs in gastric carcinoma. In the present study, we observed that mTOR and P70S6K expression were examined in gastric carcinoma, adjacent non-tumorous mucosaand adenoma, and compared with the clinicopathological parameters of tumors to explore the clinicopathological significance and molecular role of the mTOR signal pathway in the stepwise development of gastric carcinomas.

## Materials and methods

### Subjects

Gastric carcinomas (n = 421) were collected from the surgical resection, adenoma (n = 45) from endoscopic biopsy or polypectomy, and gastritis (n = 49) from the endoscopic biopsy in Shengjing Hospital of China Medical University and the First Affiliated Hospital of China Medical University between 1993 and 2006. All carcinomas were adenocarcinomas and the adenoma group was free from non-neoplastic polyp types, leiomyomas and benign GIST's. The patients with gastric carcinoma were 293 men and 126 women (29~91 years, mean = 65.4 years). Among them, 156 cases have carcinomas accompanied with lymph node metastasis. None of the patients underwent chemotherapy or radiotherapy before surgery. They all provided consent for use of tumour tissue for clinical research and our University Ethical Committee approved the research protocol. We followed up all patients by consulting their case documents or through telephone.

### Pathology

All tissues were fixed in 4% neutralised formaldehyde, embedded in paraffin and incised into 4 mm sections. These sections were stained by haematoxylin-and-eosin (HE) to confirm their histological diagnosis and other microscopic characteristics. The staging for each gastric carcinoma was evaluated according to the Union Internationale Contre le Cancer (UICC) system for the extent of tumour spread [12]. Histological architecture of gastric carcinoma was expressed in terms of Lauren's classification [13,14]. Furthermore, tumour size, depth of invasion, lymphatic and venous invasion were determined.

### Tissue microarray (TMA)

Prior to TMA construction, all tissue slides were histopathologically re-evaluated by one pathologist and. Two 2.0-mm tissue cores were taken from representative areas of gastric samples using a manual arraying device (MTA-1; Beecher Inc., Sun Prairie, WI, USA) and mounted in a new recipient block. Four-μm-thick sections were consecutively incised from the recipient block and transferred to poly-lysine-coated glass slides. HE staining was performed on TMA for confirmation of tumor tissue.

### Immunohistochemistry

For the immunohistochemical procedure, 4-μm-thick sections were deparaffinized with xylene and rehydrated through an alcohol gradient. The sections were quenched with 3% hydrogen peroxide in absolute methanol for 20 min to block endogenous peroxidase activity, and heated in a microwave for 15 min in citrate buffer (0.01 mol/L, pH 6.0) to retrieve the antigen. The sections were incubated with rabbit anti-mTOR antibody (Clone ID: Y392, 1612-1, Epitomics, USA; 1:250) and anti-phospho-p70 s6 kinase (pT389. Clone ID: E175, 1175-1, Epitomics, USA; 1:50) for 60 min, followed by exposure to the anti-rabbit Envison-PO (DAKO, USA) antibody for 60 min. Binding sites were visualized with 3, 3'-diaminobenzidine (DAB) with the 5-min reaction. After each treatment, the slides were washed with TBST (10 mM Tris-HCl, 150 mM NaCl, 0.1% Tween 20) three times for 1 min. After counterstained with Mayer's haematoxylin, the sections were dehydrated, cleared and mounted. Omission of the primary antibody was used as a negative control.

**Figure 1:**
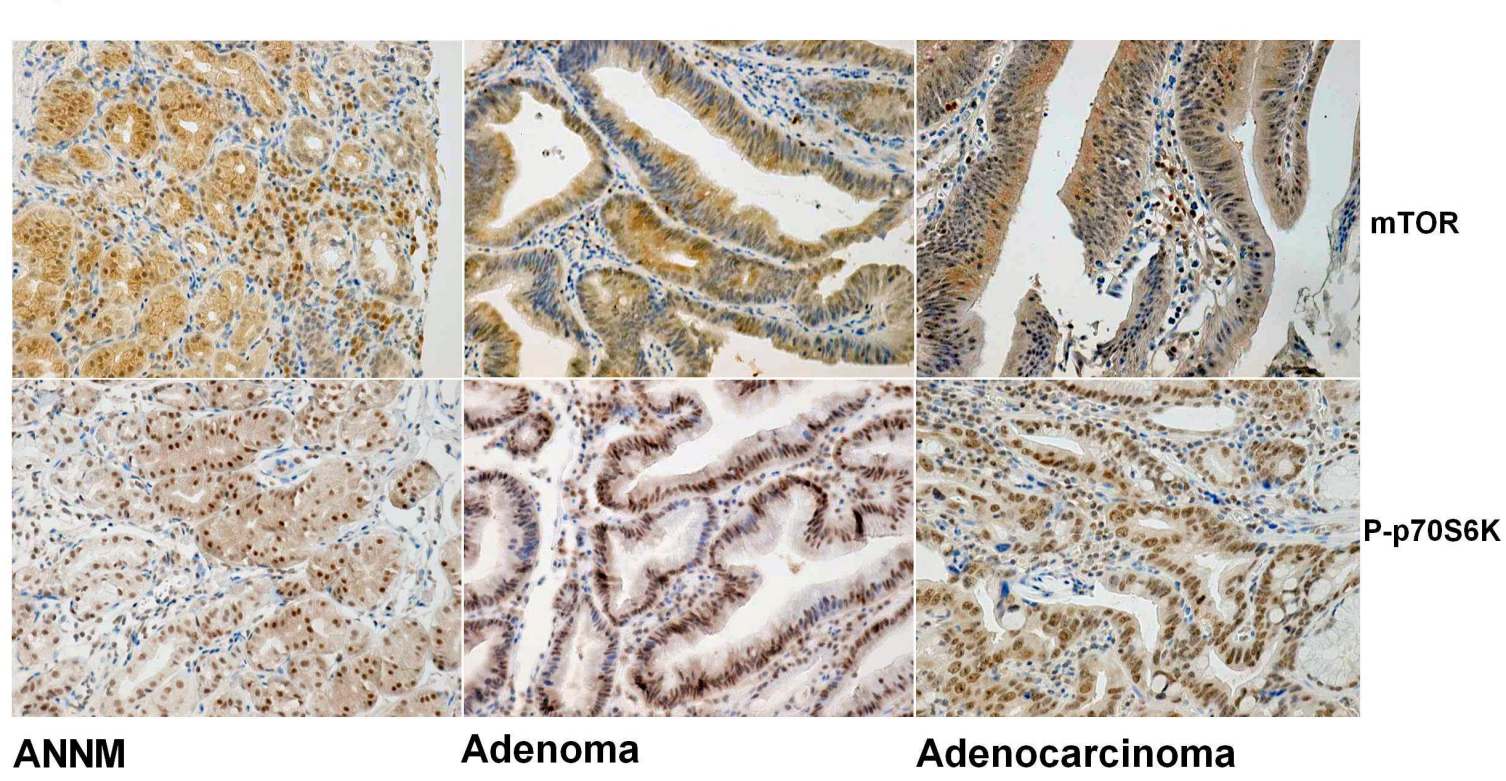
**Immunohistochemical staining in gastritis, gastric adenoma and carcinoma**. Note mTOR positivity was strongly observed in the cytoplasm, while P70S6K in the cytoplasm and nucleus. mTOR expression was observed in non-cancerous mucosa (a, +++), adenoma (b, +++) and carcinoma(c, +++). P70S6K protein was immunoreactive in non-neoplastic mucosa (d, +++), adenoma (e, +++) and carcinoma (f, +++).

As indicated in Figure 1, mTOR was positively localized in the cytoplasm, whereas P70S6K in the cytoplasm and nucleus. One hundred cells were randomly selected and counted from 5 representative fields of each section blindly by three independent observers. The positive percentage of counted cells was graded semi-quantitatively according to a four-tier scoring system: negative (-), 0~5%; weakly positive (+), 6~25%; moderately positive (++), 26~50%; and strongly positive (+++), 51~100%.

### Statistical Analysis

Statistical evaluation was performed using Spearman correlation test to analyze the rank data. Kaplan-Meier survival plots were generated and comparisons between survival curves were made with the log-rank statistic. The Cox's proportional hazards model was employed for multivariate analysis. p < 0.05 was considered as statistically significant. SPSS 10.0 software was employed to analyze all data.

## Results

### mTOR and p70 S6 kinase expression in gastric carcinomas

As showed in Figure 1, mTOR was positively immunostained in the cytoplasm of gastric epithelial cells, adenomas and carcinomas. Overall, mTOR expression was detected respectively in 66.3% of NNM (n = 197). 47 out of 67 adenoma patients (70.1%), and 255 out of total 412 gastric carcinoma patients (61.2%). Statistically, there was no significance between these three groups (p > 0.05, Table 1). As summarized in Table 2, cytoplasmic P706SK was highly expressed in adenoma (53.7%, 37/67), compared with NNM (34.5%, 68/197, p < 0.05). However, nuclear p70S6K expression was positive in 216 cases of 404 gastric carcinomas (59.5%), lower than gastric adenoma (83.6%, 56/67) and ANTMs (78.2%, 154/197, p < 0.05, Table 3)

**Table 1:** mTOR expression in gastric carcinogenesis.

Groups	N	mTOR expression				
		
		-	+	++	+++	PR(%)
Non-neoplastic mucosa	197	65	89	30	13	66.3
Adenoma	67	20	29	16	2	70.1
Carcinomas	412	157	154	78	23	61.2

PR, positive rate; p > 0.05

**Table 2:** CytoplasmicP70S6K expression in gastric carcinogenesis

Groups	N	Cytoplasmic P70S6K expression				
		
		-	+	++	+++	PR(%)
Non-neoplastic mucosa	197	129	43	24	1	34.5
Adenoma	67	30	31	5	0	53.7*
Carcinomas	394	237	115	39	3	39.8

PR, positive rate

*compared with non-neoplastic mucosa, p < 0.05

**Table 3:** Nuclear P70S6K expression in gastric carcinogenesis

Groups	N	Nuclear P70S6K expression				
		
		-	+	++	+++	PR(%)
Non-neoplastic mucosa	197	43	67	62	25	78.2
Adenoma	67	11	20	28	8	83.6
Carcinomas	404	188	123	73	20	59.5*

*compared with non-neoplastic mucosa or adenoma, p < 0.001

These three markers were preferably expressed in the older patients with gastric cancer and intestinal-type carcinoma (p < 0.05, Table 4, Table 5 and Table 6). mTOR expression was positively correlated with the cytoplasmic and nuclear expression of P70S6K (p < 0.05, Table 4). mTOR expression was inversely correlated with tumour size, depth of invasion, lymphatic invasion, lymph node metastasis and UICC staging (p < 0.05), but not with sex or venous invasion (p > 0.05, Table 4). Nuclear P70S6K expression was inversely linked to tumor size, depth of invasion, lymph node metastasis and UICC staging (p < 0.05, Table 6).

**Table 4:** Relationship between mTOR expression and clinicopathological features of gastric carcinomas

**Clinicopathological features**	**n**	**mTOR expression**					
		
		**-**	**+**	**++**	**+++**	**PR(%)**	***P *value**
Age(years)							0.042
<65	163	64	66	30	3	60.7	
≥65	249	93	88	48	20	62.7	
Sex							0.089
male	288	109	101	56	22	62.2	
Female	124	48	53	22	1	61.3	
Tumor size(cm)							0.457
<4	221	81	83	44	13	63.3	
≥4	191	76	71	34	10	60.2	
Depth of invasion							0.361
T_is-1_	222	79	86	45	12	64.4	
T_2-4_	190	78	68	33	11	58.9	
Lymphatic invasion							0.845
-	267	99	103	51	14	62.9	
+	145	58	51	27	9	60.0	
Venous invasion							0.063
-	358	140	135	66	17	60.9	
+	54	17	19	12	6	68.5	
Lymph node metastasis							0.168
-	263	90	105	55	13	65.8	
+	149	67	49	23	10	55.0	
UICC staging							0.898
0-I	234	87	90	45	12	62.8	
II-IV	178	70	64	33	11	60.7	
Lauren classification							0.000
Intestinal type	230	71	84	56	19	69.1	
Diffuse type	173	81	67	21	4	53.2	
Cytoplasmic P70S6K expression							0.000
-	207	109	72	22	4	47.3	
+~+++	151	27	57	48	19	82.1	
Nuclear P70S6K expression							0.000
-	162	95	48	15	4	41.4	
+~+++	206	39	90	58	19	81.1	

PR = positive rate; T_is _= carcinoma in situ; T_1 _= lamina propria and submucosa; T_2 _= muscularis propria and subserosa; T_3 _= exposure to serosa; T_4 _= invasion into serosa; UICC = Union Internationale Contre le Cancer

**Table 5:** Relationship between cytoplasmic P70S6K expression and clinicopathological features of gastric carcinomas

Clinicopathological features	N	Cytoplasmic P70S6K expression					
		
		-	+	++	+++	PR(%)	*P *value
Age(years)							0.001
<65	158	108	37	13	0	31.6	
≥65	236	129	78	26	3	45.3	
Sex							0.161
male	273	162	76	32	3	40.7	
Female	121	75	39	7	0	38.0	
Tumor size(cm)							0.393
<4	199	117	58	22	2	41.2	
≥4	195	120	57	17	1	38.5	
Depth of invasion							0.747
T_is-1_	197	117	59	19	2	40.6	
T_2-4_	197	120	56	20	1	39.5	
Lymphatic invasion							0.739
-	247	150	73	21	3	39.3	
+	147	87	42	18	0	40.8	
Venous invasion							0.452
-	235	202	101	29	3	56.6	
+	55	35	10	10	0	36.4	
Lymph node metastasis							0.550
-	239	140	74	23	2	41.4	
+	155	97	41	16	1	37.4	
UICC staging							0.996
0-I	213	128	63	20	2	39.9	
II-IV	181	109	52	19	1	39.8	
Lauren classification							0.000
Intestinal type	209	96	81	30	2	54.1	
Diffuse type	174	134	30	9	1	23.0	
Nuclear P70S6K expression							0.000
-	188	153	28	7	0	18.6	
+~+++	202	83	84	32	3	58.9	

PR = positive rate; T_is _= carcinoma in situ; T_1 _= lamina propria and submucosa; T_2 _= muscularis propria and subserosa; T_3 _= exposure to serosa; T_4 _= invasion into serosa; UICC = Union Internationale Contre le Cancer

**Table 6:** Relationship between nuclear P70S6K expression and clinicopathological features of gastric carcinomas

Clinicopathological features	N	Nuclear P70S6K expression					
		
		-	+	++	+++	PR(%)	*P *value
Age(years)							0.042
<65	165	86	49	20	10	47.9	
≥65	39	102	74	53	10	57.3	
Sex							0.172
male	282	127	85	54	16	55.0	
Female	122	61	38	19	4	50	
Tumor size(cm)							0.001
<4	210	86	59	52	13	59.0	
≥4	194	102	64	21	7	47.4	
Depth of invasion							0.000
T_is-1_	208	81	61	53	13	61.1	
T_2-4_	196	107	62	20	7	45.4	
Lymphatic invasion							0.171
-	257	114	77	54	12	55.6	
+	147	74	46	19	8	49.7	
Venous invasion							0.611
-	340	164	98	65	13	51.8	
+	64	24	25	8	7	62.5	
Lymph node metastasis							0.000
-	248	102	72	59	15	58.9	
+	156	86	51	14	5	44.9	
UICC staging							0.002
0-I	213	93	64	53	13	61.0	
II-IV	181	95	59	20	7	47.5	
Lauren classification							0.000
Intestinal type	221	76	70	58	17	65.6	
Diffuse type	172	105	52	12	3	40.0	

PR = positive rate; T_is _= carcinoma in situ; T_1 _= lamina propria and submucosa; T_2 _= muscularis propria and subserosa; T_3 _= exposure to serosa; T_4 _= invasion into serosa; UICC = Union Internationale Contre le Cancer

### Univariate and multivariate survival analysis

**Figure 2:**
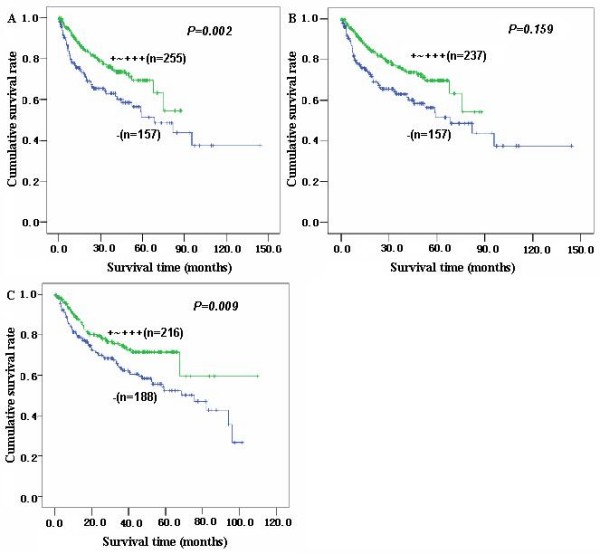
**Correlation between mTOR or p70S6K status and prognosis of the gastric carcinoma patients**. Kaplan-Meier curves for cumulative survival rate of patients with gastric carcinomas according to the mTOR(A) and cytoplasmic(B) or nuclear (C) p70S6K expression in gastric carcinomas.

Follow-up information was available on 412 gastric carcinoma patients for periods ranging from 0.2 months to 12.2 years (median = 67.3 months). The 122 patients died from carcinoma and several cases dying from other disease has been excluded. Figure 2 showed survival curves stratified according to mTOR, cytoplasmic or nuclear P70S6K expression for gastric carcinomas. Univariate analysis using the Kaplan-Meier method indicated cumulative survival rate of patients with weak, moderate or strong mTOR and nuclear p70S6K expression to be obviously higher than without its expression (p < 0.05). Multivariate analysis using Cox' s proportional hazard model indicated that age, depth of invasion, lymphatic invasion, lymph node metastasis, Lauren's classification and mTOR expression (p < 0.05), but not sex, tumor size, UICC staging, cytoplasmic or nuclear P70S6K expression were independent prognostic factors for overall gastric carcinomas (p > 0.05, Table 7).

**Table 7:** Multivariate analysis of clinicopathological variables for survival with gastric carcinomas

Clinicopathological parameters	Relative risk (95%CI)	*p *value
Age(≥ 65 years)	1.857(1.206-2.859)	0.005
Sex(male)	1.587(0.977-2.577)	0.062
Tumor size(≥ 4)	1.372(0.776-2.426)	0.277
Depth of invasion (T_2-4_)	2.793(1.323-5.898)	0.007
Lymphatic invasion(+)	2.086(1.230-3.538)	0.006
Venous invasion(+)	1.080(0.663-1.758)	0.758
Lymph node metastasis(+)	2.842(1.463-5.523)	0.002
Lauren's classification (diffuse-tape)	1.914(1.178-3.110)	0.009
mTOR (+-+++)	0.737(0.547-0.992)	0.044
Cytoplasmic P70S6K expression (+-+++)	1.061(0.765-1.472)	0.724
Nuclear P70S6K expression (+-+++)	0.854(0.625-1.166)	0.320

CI = confidence interval.

## Discussion

Mammalian target of rapamycin (mTOR) is also known as FKBP-rapamycin-associated protein or rapamycin and FKBP target and functions as a serine/threonine protein kinase to sense adenosine triphosphate and amino acids to balance nutrient availability and cell growth. When sufficient nutrients are available, mTOR is phosphorylated via the phosphoinositide 3-kinase (PI3K)/AKT signaling pathway, transmits a positive signal to p70 S6 kinase (p70S6K), and participates in the inactivation of the eukaryotic translation initiation factor 4E inhibitor, 4EBP1. Therefore, mTOR plays a key role in cellular growth and homeostasis, and its regulation is frequently altered in tumors [8,15].

Although mTOR protein can shuttle between the nucleus and cytoplasm [16,17], we only observed its cytoplasmic distribution in line with the figure of its antibody data sheet. The phenomenon might be due to cell specificity and different antibody. In the present study, the antibody was produced with a synthetic peptide corresponding to residues near the C-term of PI3K/PI4K domain of human mTOR/FRAP as an immunogen. In addition, we found no difference in mTOR expression between gastric ANTC, adenoma and carcinoma, which suggested its role of growth-regulating in all gastric epithelial and lesion cells. However, its active form, phosphorylated mTOR might contribute to the carcinogenesis according to the literature [18-23]. In contrast, its down-stream target, the aberrant expression of cytoplasmic and nuclear phoshorylated p70S6K occurred in gastric adenoma-adenocarcinoma sequence. For instance, adenoma displayed higher cytoplasmic expression of active p70S6K than NNM group, whereas nuclear counterpart was less expressed in carcinoma than NNM and adenoma. It was speculated that different subcelluar distribution of phospho-p70S6K might have distinct biological function in the malignant transformation of gastric epithelial cells.

The 70-kDa S6 kinase (p70S6K) is a cytoplasmic Ser/Thr kinase that is mainly known to regulate protein translation through phosphorylation of the 40S ribosomal protein S6. Activation of p70S6K is achieved through phosphorylation on multiple Ser/Thr residues by stimulation with growth factors such as epidermal growth factor (EGF), thrombin, and lysophosphatidic acid (LPA)[23,24]. To the role of phopsho-p0S6K protein in the progression of gastric carcinoma, its expression was compared with the aggressive behaviors of carcinoma and for the first time found that nuclear phosphor-P70S6K expression was inversely linked to tumor size, depth of invasion, lymph node metastasis and UICC staging. It was suggested that down-regulated expression of nuclear phospho-P70S6K was involved in the growth, invasion and metastasis of gastric carcinoma and might be employed to indicate the biological behaviors of gastric carcinoma in clinicopathological practice.

Although gastric cancer is malignant tumor originating from the same gastric epithelium, its morphological features vary substantially with the individual patients [13]. According to Lauren's classification, intestinal-type carcinomas are characterized by cohesive carcinoma cells forming gland-like tubular structures with expanding or infiltrative growth pattern. The cell cohesion is less apparent or absent in diffuse-type carcinoma and cancer cells diffusely spread in the gastric wall lesions. Tumors that contain approximately equal quantities of intestinal and diffuse components are called mixed carcinoma [13,14]. These three markers were preferably expressed in the older patients with gastric cancer and intestinal-type carcinoma. Here, it was noted that mTOR, cytoplasmic and nuclear P70SK6 expression was higher in intestinal-than diffuse-type carcinomas, indicating that these three markers might play an important role in intestinal-type carcinogenesis, but less in de novo carcinogenic pathway and underlie the molecular basis for differentiation of both carcinomas.

To clarify the prognostic significance of mTOR, cytoplasmic or nuclear P70S6K expression, we here analyzed their relation with the survival of 412 patients with gastric carcinoma and found a close relationship link between the positivity of mTOR and nuclear phospho-P70S6K expression and favorable survival. Multivariate analysis demonstrated six independent prognostic factors such as age, depth of invasion, lymphatic invasion, lymph node metastasis, Lauren's classification and mTOR expression were independent prognostic factors for overall gastric carcinomas. However, several evidences indicated that phosphor-mTOR expression was closely linked to the poor prognosis of the patients with cervical adenocarcioma or hepatocellular carcinoma [18,25]. PS6K overexpression was reported to correlate with worse distant disease-free survival of early-stage breast cancer patients. The paradoxical phenomenon might be attributed to the different mTOR types or different subcellular distribution of p70S6K protein. Here, nuclear p70S6K was inversely related to the tumor size, depth of invasion, lymph node metastasis and UICC staging, which are aggressive appearances of gastric cancer. The finding indicated p70 S6 phosphorylation in the nucleus might play some inhibitory role in gastric cancer and subsequent progression distinct from that in the cytoplasm.

## Conclusion

Aberrant expression of p-P70S6K might play an important role of malignant transformation of gastric epithelial cells and was closely related to growth, invasion, metastasis and prognosis of gastric carcinomas and was considered as a promising marker to indicate the pathobiological behaviors. The distinct expression of mTOR and p-P70S6K could be employed to differentiate the intestinal- and diffuse-type carcinomas and underlay the molecular mechanism about the differentiation of both carcinomas. Nuclear p-p70S6K was a good marker to indicate the favorable prognosis of gastric carcinoma patients, albeit dependent on other parameters, but mTOR expression was an independent factor for the prognosis.

## Competing interests

The authors declare that they have no competing interests.

## Authors' contributions

LX designed the research and wrote the paper. WSL and YCW carried out the the immunoassays and collected the gastric cancer tissues. LX and WSL carried out the pathological diagnosis and data analysis. YD prepared the Tissue microarray. All authors have read and approved the manuscript.
